# Supplementary material for: Cholesterol-lowering effects of oats induced by microbially produced phenolic metabolites in metabolic syndrome: a randomized controlled trial
Source: Nat Commun. 2026 Jan 14;17:598. doi: 10.1038/s41467-026-68303-9 (PMC12808737; doi:10.1038/s41467-026-68303-9)
Supplement: Supplementary file 1 — Supplementary Information [file 41467_2026_68303_MOESM1_ESM.pdf]

SUPPLEMENTARY FIGURES

Cholesterol-lowering effects of oats induced by microbially produced phenolic metabolites in metabolic syndrome: a randomized controlled trial

Klümpen L et al.

Correspondence: Marie-Christine Simon, mcsimon@uni-bonn.de

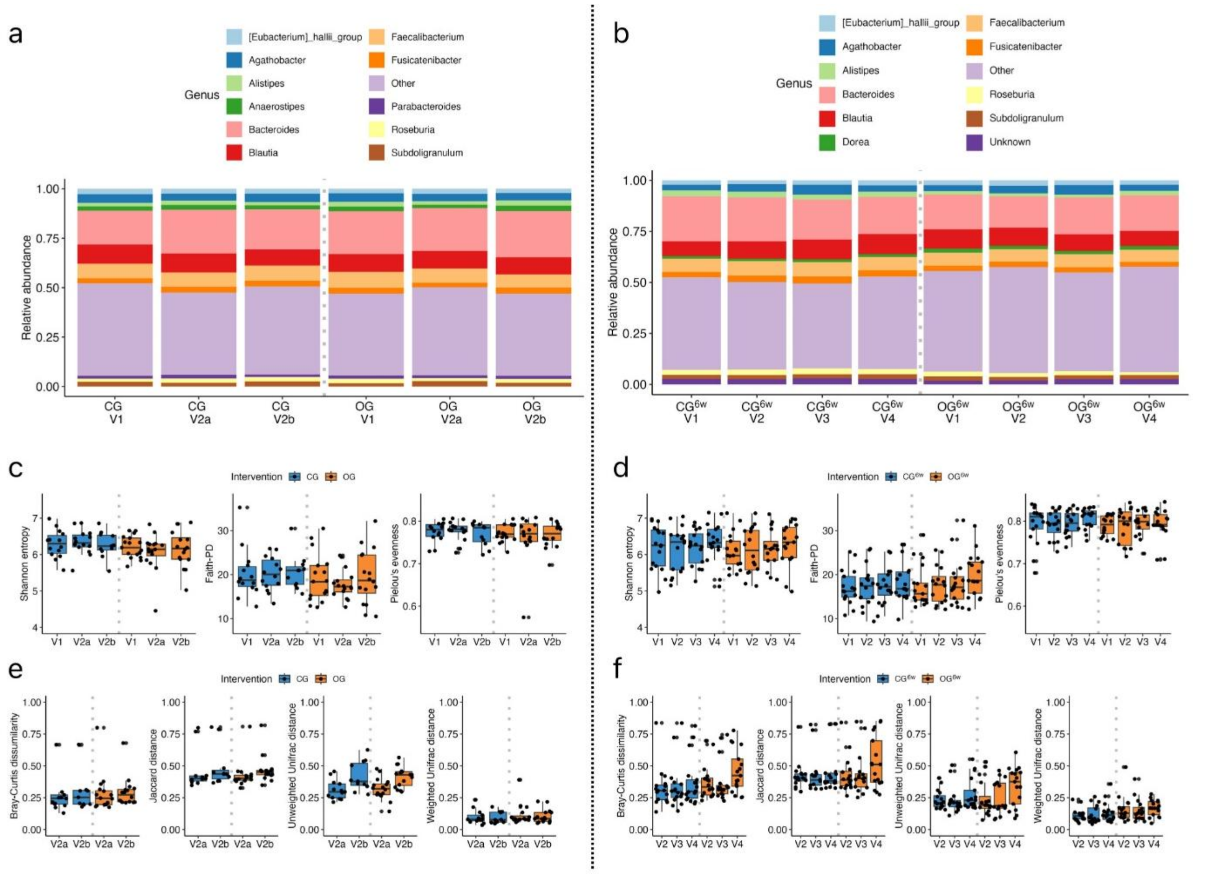

Supplementary Fig. 1 Diet-induced changes in microbial composition and diversity.

a, **b** Relative abundance of the 11 most abundant genera in the (**a)** short-term and (**b**) six-week intervention study. These genera comprised approximately 50% of the fecal microbiome, whereas the remaining 133 genera made up the remaining 50%. **c**, **d** Alpha diversity indices (Shannon entropy, Faith-PD, and Pielou's evenness) of the (**c**) short-term intervention study (OG: *n* = 17 [V1], *n* = 16 [V2a, V2b]; CG: *n* = 14 [V1, V2a], *n* = 11 [V2b]) and (**d**) the six-week intervention study (OG^{6w}: *n* = 17 [V1+V4], *n* = 16 [V2, V3]; CG^{6w}: *n* = 17 [V1+V4], *n* = 16 [V2, V3]). **e**, **f** Beta diversity metrics (Bray Curtis dissimilarity, Jaccard distance, unweighted and weighted Unifrac distance) for the (**e**) short-term intervention study (OG: *n* = 16 [V2a, V2b]; CG: *n* = 13 [V2a], *n* = 11 [V2b]) and (**f**) the six-week intervention study (OG^{6w}: *n* = 16 [V2, V3], *n* = 17 [V4]; CG^{6w}: *n* = 16 [V2, V3], *n* = 17 [V4]). **c** – **f** Diversity scores are shown per individual as black dots, as median (center line) and quartiles (box limits: Q1, Q3) for CG and CG^{6w} in blue and OG and OG^{6w} in orange (whiskers extend to 1.5x interquartile range). Abbreviations: CG, control group; CG6w, six-week control group; OG, oat group; OG6w, six-week oat group; V, visit. Source data are provided as a Source Data file.

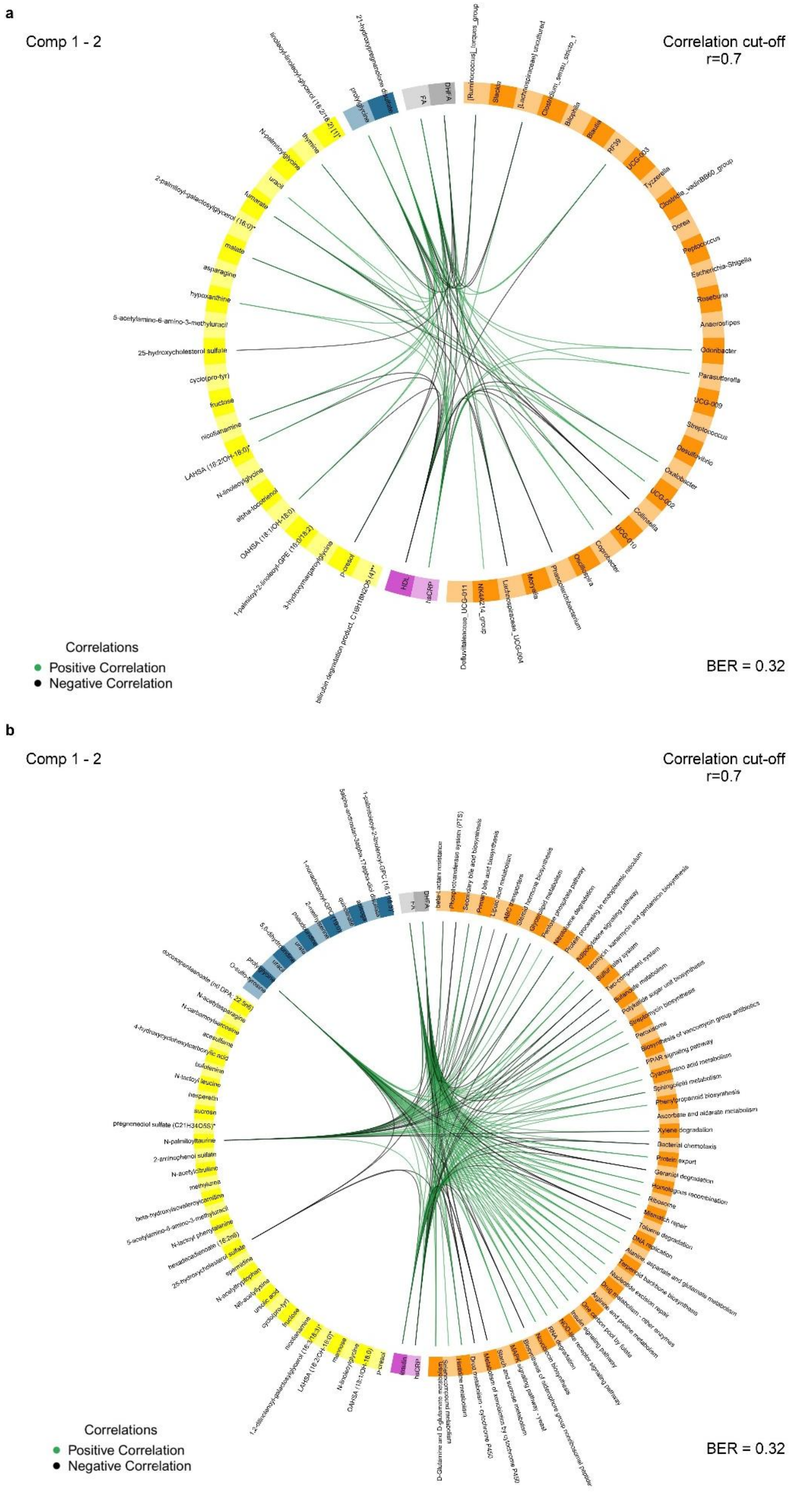

Supplementary Fig. 2 Associations between the change in clinical markers, metabolites and gut microbiota following the six-week dietary intervention.

Supplementary Fig. 2 Associations between the change in clinical markers, metabolites and gut microbiota following the six-week dietary intervention.
Circos plot shows positive (green) and negative (black) correlations (cutoff $r = 0.7$) between the selected variables in the five different data sets along component one and two, derived from the DIABLO evaluations ("models"). **a** Model 1.2 includes the following data sets: gut microbiome composition (orange), clinical marker (violet), fecal metabolites (yellow), plasma metabolites (blue), and targeted plasma metabolomic profile (DHFA, FA) (grey). **b** Model 2.2 includes the following data sets: microbial pathways (orange), clinical marker (violet), fecal metabolites (yellow), plasma metabolites (blue), and targeted plasma metabolomic profile (DHFA, FA) (grey). **a**, **b** Intra-block correlations are not presented. Log-fold change was used as the input to the models ($n = 34$ (OG^{6w}: $n = 17$), CG^{6w}: $n = 17$)). Abbreviations: BER, balanced error rate; Comp, component; DHFA, dihydroferulic acid; FA, ferulic acid; HDL, hight-density lipoprotein cholesterol; hsCRP, high-sensitivity C-reactive protein. Source data are provided as a Source Data file.

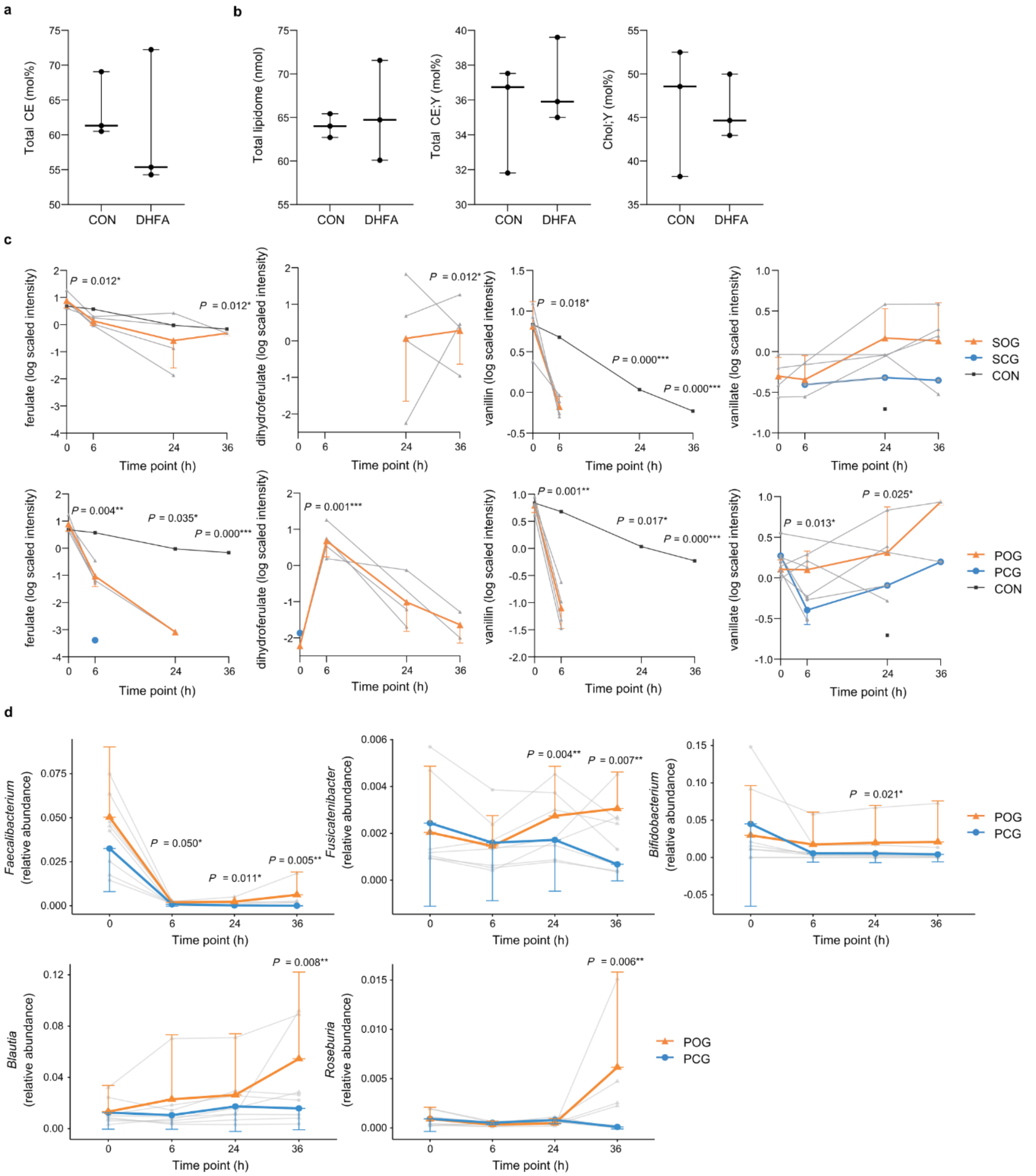

Supplementary Fig. 3 Impact of microbially produced phenolic compounds from oats on cholesterol metabolism *in vitro*.

Supplementary Fig. 3 Impact of microbially produced phenolic compounds from oats on cholesterol metabolism *in vitro*.

a, **b** Impact of DHFA on (**a**) the molar fraction (mol%) of total unlabeled cholesterol esters of the total lipidome (labeled + unlabeled) of HuH7 cells fed with13C labeled acetate and alkyne fatty acid 182 as well as on (**b**) the total lipidome (labeled + unlabeled) and the molar fraction (mol%) of alkyne cholesterol esters and alkyne cholesterol of HuH7 cells fed with an alkyne cholesterol. Samples are triplicate for each condition ($n = 3$) and presented as median and range. Differences between the treatment groups (DHFA vs. CON) were analyzed using two-sided unpaired Student's *t*-test. **c** Microbially induced changes in phenolic metabolites *in vitro* over 36 h. Data are shown as individuals data points (grey triangles: SOG and POG, grey dots: SCG and PCG; $n = 4$ each; black squares: control) and as mean ± SD (orange: SOG and POG, blue: SCG and PCG). Differences between the treatment groups were analyzed using two-sided paired Student's *t*-test with the log fold change as the input. **d** Shifts in the relative abundances of selected significant genera between POG and PCG *in vitro* over 36 h. Data are shown as individuals data points (grey triangles: POG, grey dots: PCG; $n = 4$ each) and as mean ± SD (orange: POG, blue: PCG). Differences between the groups were analyzed using two-sided paired Student's *t*-test with the fold change of the CLR-transformed count data as input (relative abundance threshold: 0.01%). Abbreviation: CON, control treatment (without DHFA); DHFA, dihydroferulic acid; PCG, physiological control group; POG, physiological oat group; SCG, starving control group; SOG, starving oat group. Source data are provided as a Source Data file.

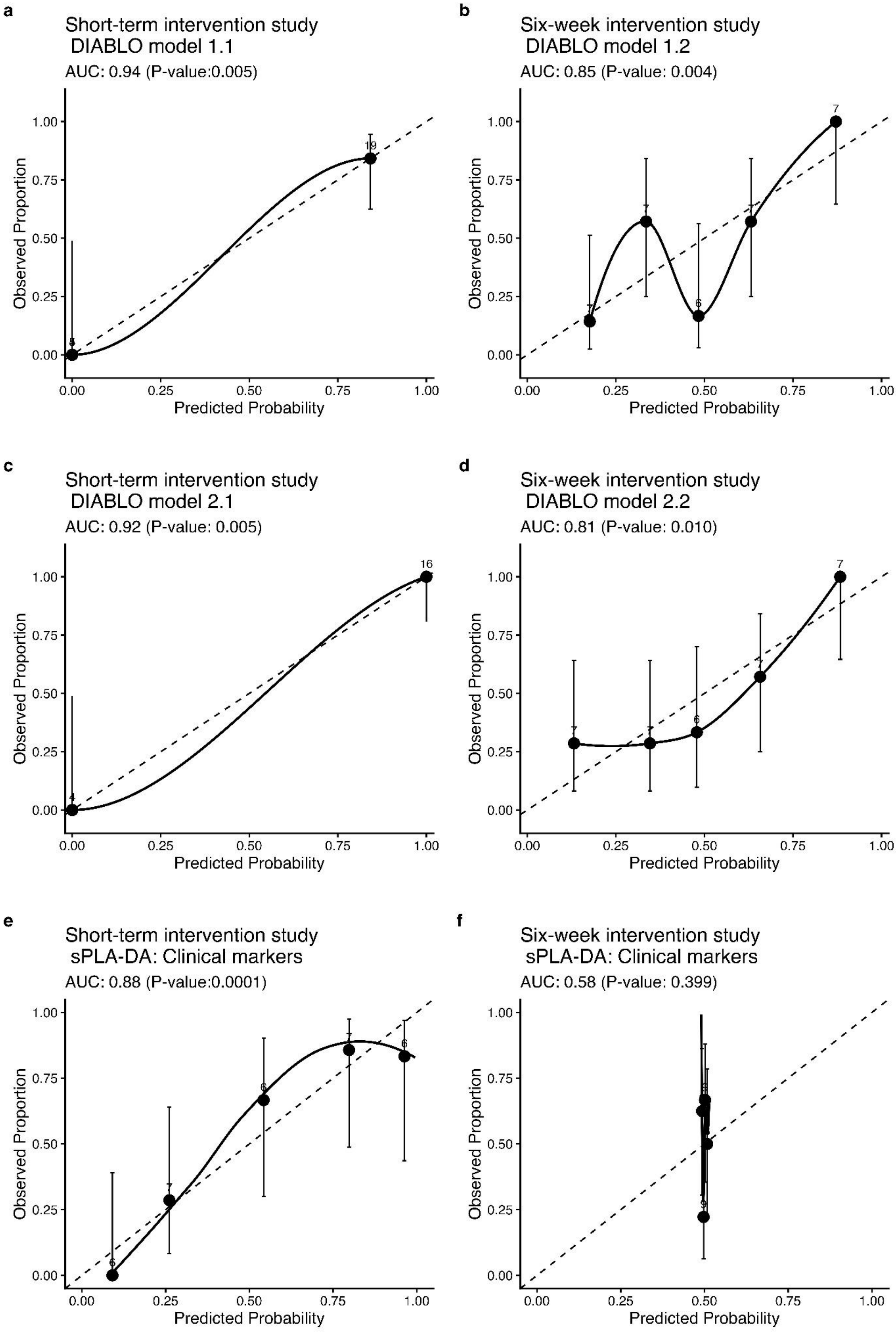

a
Short-term intervention study
DIABLO model 1.1
AUC: 0.94 (P-value:0.005)
b
Six-week intervention study
DIABLO model 1.2
AUC: 0.85 (P-value: 0.004)
c
Short-term intervention study
DIABLO model 2.1
AUC: 0.92 (P-value: 0.005)
d
Six-week intervention study
DIABLO model 2.2
AUC: 0.81 (P-value: 0.010)
e
Short-term intervention study
sPLA-DA: Clinical markers
AUC: 0.88 (P-value:0.0001)
f
Six-week intervention study
sPLA-DA: Clinical markers
AUC: 0.58 (P-value: 0.399)
Observed Proportion
Predicted Probability
0.00
0.25
0.50
0.75
1.00

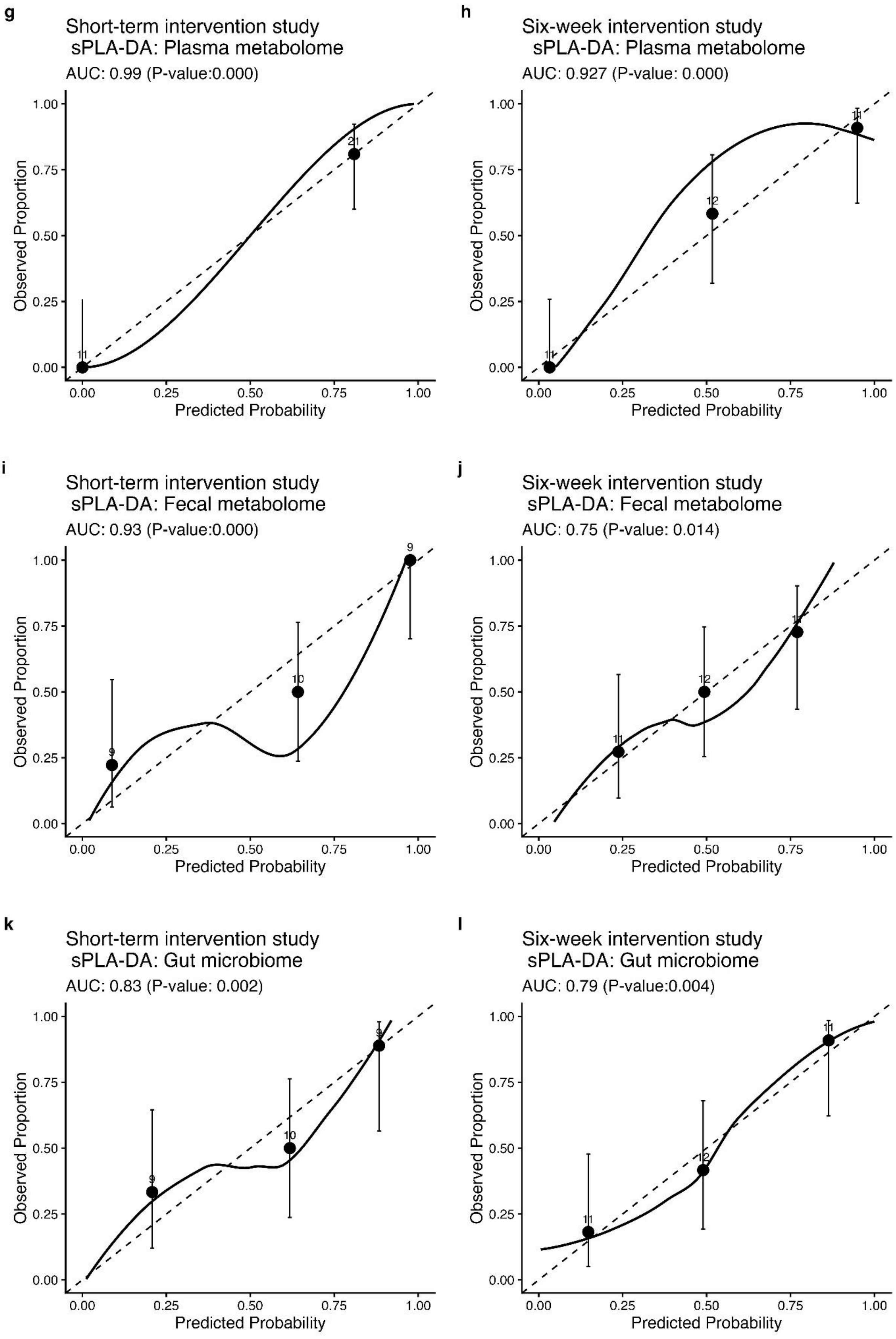
g
Short-term intervention study
sPLA-DA: Plasma metabolome
AUC: 0.99 (P-value:0.000)
Observed Proportion
Predicted Probability
h
Six-week intervention study
sPLA-DA: Plasma metabolome
AUC: 0.927 (P-value: 0.000)
Observed Proportion
Predicted Probability
i
Short-term intervention study
sPLA-DA: Fecal metabolome
AUC: 0.93 (P-value:0.000)
Observed Proportion
Predicted Probability
j
Six-week intervention study
sPLA-DA: Fecal metabolome
AUC: 0.75 (P-value: 0.014)
Observed Proportion
Predicted Probability
k
Short-term intervention study
sPLA-DA: Gut microbiome
AUC: 0.83 (P-value: 0.002)
Observed Proportion
Predicted Probability
l
Six-week intervention study
sPLA-DA: Gut microbiome
AUC: 0.79 (P-value:0.004)
Observed Proportion
Predicted Probability
0.00
0.25
0.50
0.75
1.00

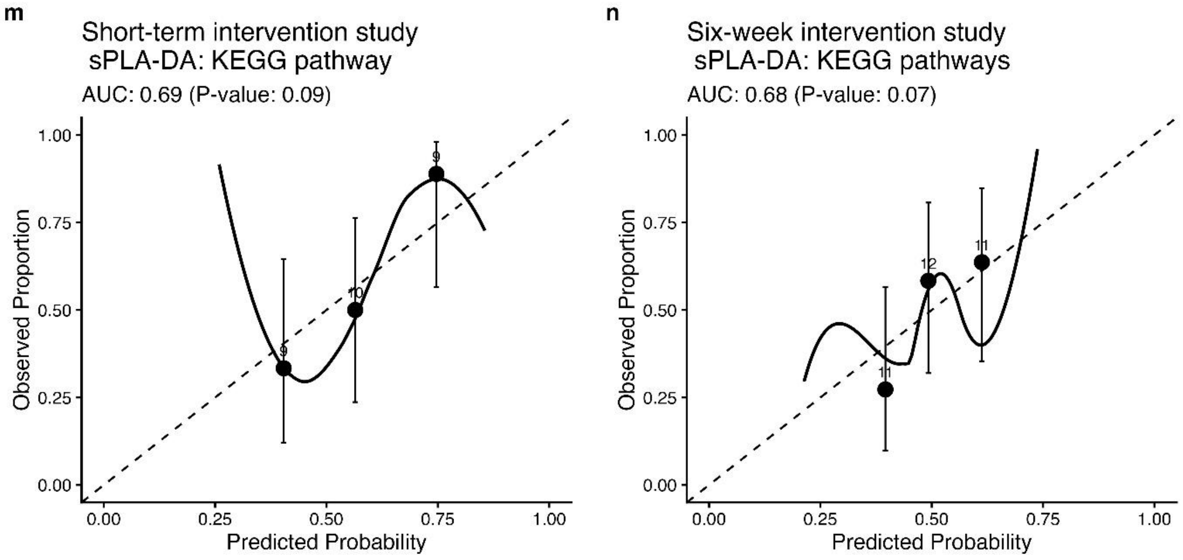

Supplementary Fig. 4 Calibration plots for all DIABLO and sPLS-DA models classifying “oats” versus “control”

a, **b** Results of the calibration analysis of the DIABLO model including the data sets gut microbiome composition, clinical markers, targeted plasma as well as global fecal and plasma metabolomic profiles for (**a**) the short-term intervention (model 1.1) and (**b**) the six-week intervention (model 1.2). **c**, **d** Results of the calibration analysis of the DIABLO model including the data sets microbial pathways, clinical markers, targeted plasma as well as global fecal and plasma metabolomic profiles for (**c**) the short-term intervention (model 2.1) and (**d**) the six-week intervention (model 2.2). **e**, **f** Results of the calibration analysis of the sPLS-DA for investigating the diet-induced modulation of metabolism following the (**e**) short-term and (**f**) six-week interventions. **g**, **h** Results of the calibration analysis of the sPLS-DA for investigating the diet-induced modulation of global plasma metabolomic profile following the (**e**) short-term and (**f**) six-week interventions. **i**, **j** Results of the calibration analysis of the sPLS-DA for investigating the diet-induced modulation of global fecal metabolomic profile following the (**e**) short-term and (**f**) six-week interventions. **k**, **l** Results of the calibration analysis of the sPLS-DA for investigating the diet-induced modulation of gut microbiome composition following the (**e**) short-term and (**f**) six-week interventions. **m**, **n** Results of the calibration analysis of the sPLS-DA for investigating the diet-induced modulation of microbial pathways following the (**e**) short-term and (**f**) six-week interventions. Calibration plots were prepared following the TRIPOD guidance (according to Moons et al. *Ann Intern Med*. 2015, Figure 8). The x-axis shows each model’s predicted probability of “oats” (from out-of-fold predictions, Platt-scaled to [0,1]); the y-axis shows the observed proportion of “oats” within probability bins. Points are bin means with 95% Wilson CIs; numbers above points indicate the number of participants in each bin. The dashed line denotes perfect calibration (y = x); the solid line is a LOESS smoother. AUC is reported above each panel. Source data are provided as a Source Data file.
