# Supplementary material for: Cholesterol-lowering effects of oats induced by microbially produced phenolic metabolites in metabolic syndrome: a randomized controlled trial
Source: Nat Commun. 2026 Jan 14;17:598. doi: 10.1038/s41467-026-68303-9 (PMC12808737; doi:10.1038/s41467-026-68303-9)
Supplement: Supplementary file 2 — Description Of Additional Supplementary File [file 41467_2026_68303_MOESM2_ESM.pdf]

Description of Additional supplementary files

Supplementary data 1:

1a) Metabolic baseline characteristics of the subjects in the short-term intervention

1b) Habitual diet of the subjects in the short-term intervention, assessed with food frequency questionnaire (FFQ)

1c) Metabolic baseline characteristics of the subjects in the six-week intervention

1d) Habitual diet of the subjects in the six-week intervention, assessed with food frequency questionnaire (FFQ)

Supplementary data 2:

2a) Energy and nutrient intake of the participants in the oat group (OG) at baseline and at the end of the follow-up period assessed by 3-day dietary records

2b) Plasma (dihydro)ferulic acid levels before and after the short-term and the six-week intervention

2c) Fasting clinical markers before and after the short-term intervention

2d) Postprandial clinical markers before and after the short-term intervention

2e) Fasting clinical markers before, during and after the six-week intervention

2f) Postprandial clinical markers before and after the six-week intervention

Supplementary data 3:

3a) To examine the correlations between changes in metabolic data, targeted plasma metabolomic pofile, global plasma and fecal metabolomic profiles, as well as gut microbiota composition following the short-term, high-dose oat diet

3b) To examine the correlations between changes in metabolic data, targeted plasma metabolomic pofile, global plasma and fecal metabolomic profiles, as well as gut microbiota function following the short-term, high-dose oat diet

3c) To examine the correlations between changes in metabolic data, targeted plasma metabolomic pofile, global plasma and fecal metabolomic profiles, as well as gut microbiota composition following the six-week, moderate oat diet

3d) To examine the correlations between changes in metabolic data, targeted plasma metabolomic pofile, global plasma and fecal metabolomic profiles, as well as gut microbiota function following the six-week, moderate oat diet

Supplementary data 4:

4a) Correlations between the change in TC and LDL-C and the change in the targeted plasma metabolomic profile (FA, DHFA) within the short-term dietary intervention

4b) Correlations between the change in TC and LDL-C and the change in the global plasma metabolomic profile within the short-term dietary intervention

4c) Correlations between the change in TC and LDL-C and the change in the global fecal metabolomic profile within the short-term dietary intervention

4d) Correlations between the change in TC and LDL-C and the change in the microbial composition (genus level) within the short-term dietary intervention

4e) Correlations between the change in TC and LDL-C and the change in the microbial function (KEGG pathways) within the short-term dietary intervention

Supplementary data 5:

5a) To identify diet-induced changes in clinical markers that distinguish OG from CG during the short-term intervention

5b) To identify diet-induced changes in clinical markers that distinguish OG6w from CG6w during the six-week intervention

5c) To identify diet-induced changes in plasma metabolomic profile that distinguish OG from CG during the short-term intervention

5d) To identify diet-induced changes in plasma metabolomic profile that distinguish OG6w from CG6w during the six-week intervention

5e) To identify diet-induced changes in fecal metabolomic profile that distinguish OG from CG during the short-term intervention

5f) To identify diet-induced changes in fecal metabolomic profile that distinguish OG6w from CG6w during the six-week intervention

5g) To identify diet-induced changes in microbial composition that distinguish OG from CG during the short-term intervention

5h) To identify diet-induced changes in microbial composition that distinguish OG6w from CG6w during the six-week intervention

5i) To identify diet-induced changes in microbial pathways that distinguish OG from CG during the short-term intervention

5j) To identify diet-induced changes in microbial pathways that distinguish OG6w from CG6w during the six-week intervention

5k) To examine the correlations between changes induced by the short-term intervention in metabolic data, targeted plasma metabolomic profile, global plasma and fecal metabolomic profiles, as well as gut microbiota composition (model 1.1)

5l) To examine the correlations between changes induced by the short-term intervention in metabolic data, targeted plasma metabolomic profile, global plasma and fecal metabolomic profile,s as well as gut microbiota function (model 2.1)

5m) To examine the correlations between changes induced by the six-week intervention in metabolic data, targeted plasma metabolomic profile, global plasma and fecal metabolomic profiles, as well as gut microbiota composition (model 1.2)

5n) To examine the correlations between changes induced by the six-week intervention in metabolic data, targeted plasma metabolomic profile, global plasma and fecal metabolomic profile,s as well as gut microbiota function (model 2.2)

Supplementary data 6:

6a) Confirmation „Metabolon Compliance with Community Standards"

6b) Minimum Reporting Standards Document – Reporting the Use of Different QC Samples in Untargeted Studies (version 1)

6c) Metabolite Reporting Checklist
