# Supplementary material for: Cholesterol-lowering effects of oats induced by microbially produced phenolic metabolites in metabolic syndrome: a randomized controlled trial
Source: Nat Commun. 2026 Jan 14;17:598. doi: 10.1038/s41467-026-68303-9 (PMC12808737; doi:10.1038/s41467-026-68303-9)
Supplement: Supplementary file 8 — Supplementary Data 6 [file 41467_2026_68303_MOESM8_ESM.pdf]

Supplementary Data 6: Additional Requirements for Metabolomics

- Confirmation „Metabolon Compliance with Community Standards”
- Minimum Reporting Standards Document – Reporting the Use of Different QC Samples in Untargeted Studies (version 1)
- Metabolite Reporting Checklist

To: Marie-Christine Simon, PhD MSc RD / Assistant Professor of Nutrition and Microbiota, Universität Bonn

From: Metabolon R&D and QA/RA

Date November 6, 2025

RE: Metabolon Compliance with Community Standards

This memo is to confirm that Metabolon's reporting of metabolomics data and methods complies with the community requirements, as outlined in Box 1 of the publication "Mass spectrometry-based metabolomics: a guide for annotation, quantification and best reporting practices" Nature Methods **18**, 747-756 (2021) (https://doi.org/10.1038/s41592-021-01197-1).

Regards,

Anne Evans, PhD / Sr. Director, Core Research & Development

Pamela Nakhle, PhD / Sr. Director, Quality Assurance & Regulatory Affairs

Minimum reporting standards document –
reporting the use of different QC samples in untargeted studies (version 1)

Authors: Metabolomics Quality Assurance & Quality Control Consortium (mQACC) Community Engagement Working group

1. Please complete this form for each different analytical platform applied in the reported study. Different LC-MS assays can be grouped together in to a single form. Different NMR assays can be grouped together in to a single form.
2. If multiple different types of QC samples are used then list all in this form.
3. Definitions for different QC sample types is available at the end of the document

Questions

Q1. Which analytical platform(s) was applied in this study?

- NMR spectroscopy ☐
- GC-MS ☐
- LC-MS ☑
- CE-MS ☐
- DIMS ☐
- IR/Raman spectroscopy ☐
- Other ☐

Q2. Was a system suitability sample or suitability sample used during the reported study and is its composition and acceptance criteria reported in the manuscript?

YES ☑

NO ☐

NOT APPLICABLE ☐

Q3. Were internal standards used during the reported study and are the composition and acceptance criteria reported in the manuscript? For NMR, was an alternative calibration method, such as ERECTIC, used?

YES ☑

NO ☐

NOT APPLICABLE ☐

Q4. Were blank samples used during the reported study and are the composition and acceptance criteria reported in the manuscript?

YES ☑

NO ☐

Q5. Were one or multiple types of pooled QC samples used during the reported study and are the composition and acceptance criteria reported in the manuscript?

YES ☑

NO ☐

NOT APPLICABLE

Q6. Were sample collection, storage and thawing processes reported in the manuscript?

YES ☑

NO ☐

Q7. Were the order for sample preparation and data collection of biological samples randomised and is this reported in the manuscript?

YES ☑

NO ☐

Q8. Are the QC sample data available in a metabolomics data repository (e.g. MetaboLights or Metabolomics Workbench)?

YES ☐

NO ☑

NOT APPLICABLE ☐

Definitions

1. A ***system suitability test (SST)*** sample is a solution containing a small number of authentic chemical standards (typically five to ten analytes) from which the acquired data can be quickly assessed for accuracy and precision in an automated computational approach. For LC-MS and

GC-MS the analytes are dissolved in a chromatographically suitable diluent and not in a sample matrix.

2. ***Internal standards*** are compounds of predetermined concentrations and which are representative of the metabolite classes in the test sample metabolome and which are typically included in assays performed on mass spectrometry platforms. One or multiple internal standards are added to each test sample at the same concentration to allow monitoring of accuracy and precision for *m/z*, retention time, chromatographic peak shape, and peak area for every biological test sample.

3. Different types of ***blank samples*** can be analysed and include process/extraction blanks and solvent blanks. Process blank samples/extraction blank samples are samples which have passed through the sample preparation process in the same way as a biological sample but with no biological sample included. Solvent blank samples are samples which are composed of a solvent but which has not passed through the extraction process.

4. Different types of ***pooled QC samples*** can be analysed and include intra-study QC samples, intra-laboratory QC samples and inter-laboratory QC samples. Intra-study QC samples are prepared using biological samples analysed only in the study being reported. Intra-laboratory QC samples are pooled QC samples or a relevant material (e.g. a standard reference material) which are analysed in all studies within a single laboratory. Inter-laboratory QC samples are pooled QC samples or a relevant material (e.g. a standard reference material) which are analysed in single/all studies in different laboratories.

Metabolite Reporting Checklist

Level	Aspect	Information	Fill in
general aspect	Type of metabolome analysis	targeted metabolite analysis	false
		non-targeted metabolite class scale profiling	false
		non-targeted metabolome scale profiling	true
		non-targeted finger printing of mass features	false
	Type of quantification	absolute or quantification	relative quanitification
	Type of reference samples	chemcially defined	Process blank = aliquot of water taken through entire workflow
		biologically defined	Plasma: In-house Human Plasma Ref Material purchased from BioIVT, 5 replicates per 34 experimental samples. Feces: Pooled QC sample of aliquots of experimental fecal material were run with 4 replicates ever 34 samples, and 1 in-house human plasma sample ref material purchased from BioIVT.
	Type of replication	analytical (same analytical sample preparation)	Internal standards spiked into each sample prior to sample analysis were used to assess analytical variability
		technological (same biological preparation)	Technical replicates of a pooled plasma or fecal sample were run with 5 replicates per 34 experimental samples to assess whole workflow variability
		biological (same experimental condition)	no replicates of experimental samples, reruns were performed if sample failed to pass QC acceptance criteria
		full experiment	Only if batch failed to pass QC acceptance criteria, was a batch of samples reanalyzed
	Type of technology	reference publication	https://doi.org/10.1093/jalm/jfz026
	Sample preparation	method of sample preparation	chemically non-modified, organic solvent with standards used to crash proteins and other macromolecules. Supernatant is aliquoted, dried, then reconsistuted in method appropriate solvents with additional standards.
		method of chromatography/separation	UHPLC RP separation using C18 BEH column, UPLC HILIC separation using Amide BEH column
		method of ionization	Positive and negative ion HESI
		method of detection	HRAM MS - Thermo orbitrap MS - Q-Exactive
metabolite/ mass feature	Metabolite	metabolite name	Identified many compounds (see compound list for all names)
		metabolite sum formula	Identified many compounds
		metabolite structure and public source of metabolite identifier	Each metabolite identified contains references to HMDB, KEGG, and PUBCHEM, where available
	Identification	identification process	Automated with QC review of all identifications and peak alignment
		by authentic mass isotopomer added to one or all biological sample(s)	false
		by authentic reference compound within a co-processed reference mixture	false
		by authentic reference compound previously mapped to the analytical system	true
		reference library	In-house library of 5400 compounds created from the purchase of authentic standard
		type of mass spectrum	HRAM MS and MS/MS alternating scans
		by match of molecular mass (single mass fragment)	true
		by match of fragments	false
		by match of fragmentation pattern	true
		by match of mass spectrum to reference library	true
		type of retention index	true
		by match of retention time (index) to reference library	true
	Quantification	type of quantification	relative quanitification
	Validity testing	Recovery testing (chemical analog)	Standards added prior to sample extraction to monitor for reproducible recovery and solvent transfers
		Recovery testing (internally added mass isotopomer)	false
		Recovery testing (mixture of most divergent samples from the experiment)	false
		Test for linear range	Done during method validation, but not during experimental sample analysis
		Limit of quantification (LOQ)	false
		Limit of detection (LOD)	Done during method validation, but not during experimental sample analysis
