# Supplementary material for: Cholesterol-lowering effects of oats induced by microbially produced phenolic metabolites in metabolic syndrome: a randomized controlled trial
Source: Nat Commun. 2026 Jan 14;17:598. doi: 10.1038/s41467-026-68303-9 (PMC12808737; doi:10.1038/s41467-026-68303-9)
Supplement: Supplementary file 9 — Reporting Summary [file 41467_2026_68303_MOESM9_ESM.pdf]

nature portfolio

Corresponding author(s): Prof. Dr. Marie-Christine Simon

Last updated by author(s): Nov 8, 2025

Reporting Summary

Nature Portfolio wishes to improve the reproducibility of the work that we publish. This form provides structure for consistency and transparency in reporting. For further information on Nature Portfolio policies, see our Editorial Policies and the Editorial Policy Checklist.

Statistics

For all statistical analyses, confirm that the following items are present in the figure legend, table legend, main text, or Methods section.

n/a	Confirmed	
☐	☒	The exact sample size (*n*) for each experimental group/condition, given as a discrete number and unit of measurement
☐	☒	A statement on whether measurements were taken from distinct samples or whether the same sample was measured repeatedly
☐	☒	The statistical test(s) used AND whether they are one- or two-sided<br>*Only common tests should be described solely by name; describe more complex techniques in the Methods section.*
☐	☒	A description of all covariates tested
☐	☒	A description of any assumptions or corrections, such as tests of normality and adjustment for multiple comparisons
☐	☒	A full description of the statistical parameters including central tendency (e.g. means) or other basic estimates (e.g. regression coefficient) AND variation (e.g. standard deviation) or associated estimates of uncertainty (e.g. confidence intervals)
☐	☒	For null hypothesis testing, the test statistic (e.g. *F*, *t*, *r*) with confidence intervals, effect sizes, degrees of freedom and *P* value noted<br>*Give P values as exact values whenever suitable.*
☒	☐	For Bayesian analysis, information on the choice of priors and Markov chain Monte Carlo settings
☒	☐	For hierarchical and complex designs, identification of the appropriate level for tests and full reporting of outcomes
☐	☒	Estimates of effect sizes (e.g. Cohen's *d*, Pearson's *r*), indicating how they were calculated

Our web collection on statistics for biologists contains articles on many of the points above.

Software and code

Policy information about availability of computer code

Data collection	No software was used.
Data analysis	- SILVA database, 138.1 SSU Ref NR 99<br>- Qiime2 version 2021.4<br>- PICRUSt version 2.5.1<br>- SPSS (version 29.0; IBM Crop., Chicago, IL, USA)<br>- R (version 3.6.2; Boston, MA, USA): sPLS-DA (mixOmics v6.8.5 R package), PLS regression models (mxiOmics v. 4.2.2 R package)<br>- Python (version 3.10)<br>- GraphPad Prism (version 10; GraphPad Software, San Diego, CA, USA) (only for creating figures)<br>- EBISpro, based on the German nutrient database Bundeslebensmittelschlüssel, version 2016 (Max Rubner-Institut, Karlsruhe, Germany)

For manuscripts utilizing custom algorithms or software that are central to the research but not yet described in published literature, software must be made available to editors and reviewers. We strongly encourage code deposition in a community repository (e.g. GitHub). See the Nature Portfolio guidelines for submitting code & software for further information.

Data

Policy information about availability of data

All manuscripts must include a data availability statement. This statement should provide the following information, where applicable:

- Accession codes, unique identifiers, or web links for publicly available datasets
- A description of any restrictions on data availability
- For clinical datasets or third party data, please ensure that the statement adheres to our policy

The raw microbiome 16S rRNA data and the metabolomic data (global metabolomic profiles) generated in this study have been deposited in the open repository Zenodo (https://doi.org/10.5281/zenodo.16312602). These data and the de-identified personal data that support the findings of this study are not openly available due to reasons of sensitivity and are available from the corresponding author. These data are protected and are not openly available due to data privacy laws. Access to the data and additional, related documents can be obtained by contacting the corresponding author of the paper (Jun. Prof. Dr. Marie-Christine Simon, e-mail address: mcsimon@uni-bonn.de) and signing a data-sharing agreement. A response to requests for data access might be expected within two months, and the data will be available for one year after signing the data-sharing agreement.
Source data are provided with this paper.

Research involving human participants, their data, or biological material

Policy information about studies with human participants or human data. See also policy information about sex, gender (identity/presentation), and sexual orientation and race, ethnicity and racism.

Reporting on sex and gender	sex and gender were determined based on self-reporting (questionnaire); the term sex (biological atrribtute) was used in the manuscript; women (short-term intervention: n = 17 (50%), six-week intervention n = 19 (56%), in total: n = 36) and men (short-term intervention: n = 17 (50%), six-week intervention: n = 15 (44%), in total: n = 32) were included in the study with an equal distribution; statistical analysis were adjusted for sex
Reporting on race, ethnicity, or other socially relevant groupings	Participants were recruited in the surrounding area of Bonn, Germany. The study does not use the constructs of race, ethnicity, or other socially relevant groupings.
Population characteristics	Participants included in this study were men and women aged between 45 and 70 years with metabolic syndrome (BMI 27-39.9 kg/m², central obesity plus at least to further risk factors: elevated blood pressure, impaired glucose metabolism (hyperglycemia, insulin resistance), dyslipidemia (triglyceridemia, low HDL-cholesterol).<br>Within the short-term dietary intervention study, 32 participants were considered for the final analysis (intervention group: n = 17, control group: n = 15) which included 15 males and 17 females aged 59.0 ± 7.5 years (mean ± standard deviation (SD)) with a body mass index (BMI) of 32.0 ± 3.3 kg/m². All participants showed central obesity (100 %) and at least two further metabolic syndrome traits including increased blood pressure (100 %), impaired glucose metabolism (75 %) and dyslipidemia (63 %).<br>Within the six-week dietary intervention study, 34 participants were considered for the final analysis (intervention group: n = 17, control group: n = 19) which included 15 males and 17 females aged 59.7 ± 7.6 years (mean ± standard deviation (SD)) with a body mass index (BMI) of 31.6 ± 3.2 kg/m². All participants showed central obesity (100 %) and at least two further metabolic syndrome traits at the screening visit. Thus, 97 % of the subjects had elevated blood pressure, 76 % impaired glucose metabolism and 59 % dyslipidemia at baseline.
Recruitment	Participants were recruited through newspaper advertisements, flyers, and social media in the surrounding area of Bonn, Germany.
Ethics oversight	Ethics committee of the Medical Faculty, University of Bonn, Germany (Approval Number: 212/20). Written informed consent was obtained from all the participants.

Note that full information on the approval of the study protocol must also be provided in the manuscript.

Field-specific reporting

Please select the one below that is the best fit for your research. If you are not sure, read the appropriate sections before making your selection.

☒ Life sciences ☐ Behavioural & social sciences ☐ Ecological, evolutionary & environmental sciences

For a reference copy of the document with all sections, see nature.com/documents/nr-reporting-summary-flat.pdf

Life sciences study design

All studies must disclose on these points even when the disclosure is negative.

Sample size	Sample size (n = 17 participants per group, n = 68 in total) was calculated based on data from a previous intervention study that successfully assessed the effect of whole grain intake on blood dihydroferulic acid (DHFA) concentration (Vitaglione et al. AJCN. 2015), expecting a 2-fold change in the plasma DHFA concentration between the two diet groups of each intervention study using a two-sided t-test at a 5 %

	significance level and with 95 % power.
Data exclusions	Within in the human intervention study, no data were excluded from the analyses.
Replication	Anthropometric data (e.g. waist circumference and office blood pressure) were measured in duplicates as part of standardized routine measurements. The cell culture experiments were performed in triplicates. All attempts at replication were successful, especially the statistical analysis was performed by two independent researchers and the results could be reproduced.
Randomization	After the first visit, participants were randomly assigned to the experimental group or the control group of each intervention study using computer-generated randomization tables in a block format with variable block length generated by a researcher not clinically involved in the study.
Blinding	Within the human intervention study, the food item itself, here in particular oat flakes, was unable to blind, and was not intended in this particular study design. The randomization tables were concealed from the researchers and participants until the interventions were assigned by study personnel who were not involved in the data analysis. Within the in vitro batch and cell culture experiments, the experimentator cannot perform the experiment if they do not know what the samples contain. All data were analyzed by a person different from the experimentator.

Reporting for specific materials, systems and methods

We require information from authors about some types of materials, experimental systems and methods used in many studies. Here, indicate whether each material, system or method listed is relevant to your study. If you are not sure if a list item applies to your research, read the appropriate section before selecting a response.

Materials & experimental systems

n/a	Involved in the study	
☒	☐	Antibodies
☐	☒	Eukaryotic cell lines
☒	☐	Palaeontology and archaeology
☒	☐	Animals and other organisms
☐	☒	Clinical data
☒	☐	Dual use research of concern
☒	☐	Plants

Methods

n/a	Involved in the study	
☒	☐	ChIP-seq
☒	☐	Flow cytometry
☒	☐	MRI-based neuroimaging

Eukaryotic cell lines

Policy information about cell lines and Sex and Gender in Research

Cell line source(s)	Human hepatoma cell line: HuH-7 cells (JCRB0403)
Authentication	The cell line was obtained from JCRB cell bank, and we did not perform any further authentication procedure.
Mycoplasma contamination	The cell line was not tested for Mycoplasma contamination.
Commonly misidentified lines (See ICLAC register)	None.

Clinical data

Policy information about clinical studies

All manuscripts should comply with the ICMJE guidelines for publication of clinical research and a completed CONSORT checklist must be included with all submissions.

Clinical trial registration	DRKS00022169 (German Clinical Trials Register; http://www.drks.de)
Study protocol	Study protocol has been shared with Editorial Office. Access can be requested by contacting the corresponding author of the paper (Jun. Prof. Dr. Marie-Christine Simon, e-mail address: mcsimon@uni-bonn.de).
Data collection	This study comprised two 1:1 randomized, controlled, prospective dietary interventions, each with a parallel design, conducted between September 2020 and July 2022 at the Department of Nutrition and Microbiota, University of Bonn, Germany. The short-term intervention study was completed on schedule, while the six-week intervention study was delayed due to the COVID-19 pandemic.
Outcomes	Primary outcome:<br>- plasma (dihydro)ferulic acid concentration: generated by Metabolon Inc. (Morrisville) using LC-MS/MS according to Metabolon Method TAM223<br><br>Secondary outcomes:

- Anthropometric measurements (body weight, body height, waist circumference) were performed following previously published standard operative procedure. Body composition (fat mass and fat-free mass) was determined by air-displacement plethysmography using a BOD-POD body composition system (Cosmed, Firdolfingen, Germany).
- Clinical blood parameters (glucose and lipid metabolism, liver and kidney function, clinical biochemistry, inflammation status): routine laboratory analyses performed by a certified medical laboratory (Central Laboratory of the Institute of Clinical Chemistry and Clinical Pharmacology at the University Hospital Bonn, Germany) using the Roche/Hitachi Cobas c system (Roche Diagnostics, Mannheim, Germany. Serum non-esterified fatty acid (NEFA) concentrations were analyzed using an in-vitro enzymatic colorimetric method assay (NEFA-HR(2), Wako Diagnostics, Mountain View, CA, USA).
- Global metabolomic profiles: For both fasting plasma and fecal samples, non-targeted global metabolomic profiles were generated by Metabolon Inc. (Research Triangle) using UPLC-MS/MS, as previously described (Ford et al. JALM 2020)
- Gut microbiome: High-throughput 16S rRNA V3/V4 gene sequencing of the fecal microbiome was performed at Life & Brain GmbH (Bonn, Germany).

Plants

Seed stocks

Report on the source of all seed stocks or other plant material used. If applicable, state the seed stock centre and catalogue number. If plant specimens were collected from the field, describe the collection location, date and sampling procedures.

Novel plant genotypes

Describe the methods by which all novel plant genotypes were produced. This includes those generated by transgenic approaches, gene editing, chemical/radiation-based mutagenesis and hybridization. For transgenic lines, describe the transformation method, the number of independent lines analyzed and the generation upon which experiments were performed. For gene-edited lines, describe the editor used, the endogenous sequence targeted for editing, the targeting guide RNA sequence (if applicable) and how the editor was applied.

Authentication

Describe any authentication procedures for each seed stock used or novel genotype generated. Describe any experiments used to assess the effect of a mutation and, where applicable, how potential secondary effects (e.g. second site T-DNA insertions, mosiacism, off-target gene editing) were examined.
